# Interim analysis of the REASSURE (Radium-223 alpha Emitter Agent in non-intervention Safety Study in mCRPC popUlation for long-teRm Evaluation) study: patient characteristics and safety according to prior use of chemotherapy in routine clinical practice

**DOI:** 10.1007/s00259-019-4261-y

**Published:** 2019-01-12

**Authors:** Sabina Dizdarevic, Peter Meidahl Petersen, Markus Essler, Annibale Versari, Jean-Cyril Bourre, Christian la Fougère, Riccardo Valdagni, Giovanni Paganelli, Samer Ezziddin, Ján Kalinovský, Inga Bayh, Yong Du

**Affiliations:** 1 Department of Imaging and Nuclear Medicine, Royal Sussex County Hospital, Brighton and Sussex University Hospitals NHS Trust, Eastern Road, Brighton, BN2 5BE UK; 2 Brighton and Sussex Medical School, University of Brighton and Sussex, Brighton, UK; 3 Rigshospitalet, Copenhagen, Denmark; 4 Universitätsklinikum Bonn, Bonn, Germany; 5 Azienda Unità Sanitaria Locale – IRCCS di, Reggio Emilia, Italy; 6 Centre Hospitalier Métropole Savoie, Chambery, France; 7 Universitätsklinikum Tübingen, Tübingen, Germany; 8 Università degli Studi di Milano and Fondazione IRCCS Istituto Nazionale dei Tumori, Milan, Italy; 9 Istituto Scientifico Romagnolo per lo Studio e la Cura dei Tumori, Meldola, Italy; 10 Universitätsklinikum des Saarlandes, Homburg, Germany; 11 Bayer, Basel, Switzerland; 12 Bayer AG, Wuppertal, Germany; 13 Royal Marsden Hospital, London, UK

**Keywords:** Metastatic prostate cancer, Radium-223, Docetaxel, Cabazitaxel, Treatment sequence, Current practice

## Abstract

**Purpose:**

REASSURE is a global, prospective, non-interventional study to assess long-term safety of radium-223 in patients with bone metastatic castration-resistant prostate cancer. Here we report an interim analysis of patients according to previous use of chemotherapy.

**Methods:**

Radium-223 was administered in routine clinical practice. Interim safety analysis was planned after enrolment of the first 600 patients. Patient characteristics and safety data by previous administration of chemotherapy (docetaxel and/or cabazitaxel) were investigated.

**Results:**

This interim analysis included 583 patients. Median duration of observation was 7 months (range, 0–20). Nineteen patients treated with concomitant chemotherapy were excluded, 564 (97%) were eligible for exploratory analysis according to prior use of chemotherapy; 190 (34%) had previously received and completed chemotherapy, and 374 (66%) had not. In the prior versus no prior chemotherapy group, a higher proportion of patients had an Eastern Cooperative Oncology Group performance status of ≥2 (22% vs 11%) and > 20 metastatic lesions (26% vs 15%), median alkaline phosphatase (162.0 vs 115.0 U/L) and prostate-specific antigen (132.0 vs 40.2 ng/mL) levels were higher, and a lower proportion completed 6 radium-223 injections (45% vs 63%). Drug-related treatment-emergent adverse events (TEAEs) occurred in 63 and 48%, and haematological drug-related TEAEs in 21 and 9% of patients who had or had not previously received chemotherapy. Four drug-related deaths were reported, all in the prior chemotherapy group.

**Conclusions:**

The short-term safety profile of radium-223 in routine clinical practice was comparable to other clinical studies, irrespective of prior chemotherapy use. Haematological TEAEs occurred more frequently in the prior chemotherapy group, presumably due to decreased bone marrow function as a consequence of more advanced disease and prior exposure to cytotoxic therapy. Patients who had not previously received chemotherapy appeared to have a lower burden of disease at baseline, and a lower proportion discontinued radium-223 treatment.

**Electronic supplementary material:**

The online version of this article (10.1007/s00259-019-4261-y) contains supplementary material, which is available to authorized users.

## Introduction

Radium 223 dichloride (radium-223), a first-in-class, bone seeking, targeted alpha therapy, is used in the treatment of patients with mCRPC and symptomatic bone metastases. This followed reported prolonged overall survival and a favourable safety profile in patients treated with best standard of care (BSoC) and radium-223 compared with BSoC and placebo in the ALSYMPCA study [1].

Docetaxel with androgen deprivation therapy is used for the treatment of patients with metastatic hormone-sensitive prostate cancer [2–4]. Chemotherapy (docetaxel and cabazitaxel) may also be used for treating patients with mCRPC [4–6]. Whilst there is a survival benefit for patients with mCRPC receiving chemotherapy, treatment can be associated with toxic effects, including myelosuppression, and many patients are not healthy enough to receive chemotherapy or else may decline such treatment [7].

There are currently no level 1 data to guide the optimal sequencing of chemotherapy and radium-223 in patients with mCRPC. In the ALSYMPCA study a pre-specified subgroup analysis showed that radium-223 compared with placebo was found to be effective and well tolerated irrespective of previous docetaxel use [7]. Further exploratory analysis from the phase 3 study suggested that chemotherapy appeared to be well tolerated when given after radium-223 in patients with CRPC and symptomatic bone metastases [8]. Therefore, it is important to assess the safety of radium-223 in the setting of routine clinical practice, both in patients who have previously been treated with chemotherapy and in those who have not. In addition, identifying patient and disease characteristics that could select those most able to tolerate full scheduled treatment with radium-223 may optimise treatment outcome with this agent and facilitate effective sequencing with other life-prolonging therapies.

REASSURE is a prospective, global non-interventional study that recruited patients with mCRPC who were to be treated with radium-223 in routine clinical practice. The primary aim of REASSURE is to investigate long-term safety (including the incidence of second primary malignancies) over a 7-year period. The REASSURE study has allowed the collection of a substantial data set that reflects the current treatment paradigm for mCRPC and offers important insights into current routine clinical practice as we seek to maximise our opportunity to improve patient survival. Here we report an analysis of patient characteristics and safety data from the first planned interim analysis of REASSURE in patients grouped by previous administration of chemotherapy. The incidence of second primary malignancies will be reported after a longer duration of follow-up.

## Patients and methods

### Study design and treatment

The REASSURE study is a prospective, non-interventional study that planned to enrol patients from at least 150 sites in North America, Latin America, Europe, and Israel. Eligible patients had histologically or cytologically confirmed castration-resistant adenocarcinoma of the prostate with bone metastases and no known visceral metastases, and had provided signed informed consent. Patients previously treated with radium-223, those currently participating in any clinical trial, or patients for whom systemic treatment with other radiopharmaceuticals for any indication was planned, were excluded from the study.

The decision to treat with radium-223, agreed between the physician and the patient was made within current practice, independently from, and prior to, the provision of study information.

Radium-223 treatment was prescribed and administered as part of usual clinical practice and according to the locally approved label.

The study is registered with ClinicalTrials.gov NCT02141438.

### Assessments

The primary endpoints of the REASSURE study are the incidence of second primary malignancies, the incidence of treatment-emergent serious adverse events (SAEs), drug-related treatment-emergent adverse events (TEAEs), drug-related SAEs (up to 7 years after the last administration), and bone marrow suppression. Adverse events occurring from radium-223 initiation through 30 days after the last radium-223 injection were classified as treatment-emergent. Adverse events were coded using the Medical Dictionary for Regulatory Activities (MedDRA), version 19.0, and graded by the National Cancer Institute-Common Terminology Criteria for Adverse Events (NCI-CTCAE), version 4.03. Haematological toxicity was defined by the following adverse events (MedDRA preferred terms): anaemia (anaemia, haemoglobin decreased, and red blood cell count decreased); thrombocytopenia (thrombocytopenia and platelet count decreased); leukopenia (leukopenia and white blood cell count decreased); lymphopenia, (lymphopenia and lymphocyte count decreased), and neutropenia (neutropenia and neutrophil count decreased), pancytopenia, and bone marrow failure. Bone marrow suppression was indicated by the use of therapeutic or prevention measures (blood transfusion/erythropoietin/colony growth-stimulating factors) up to 6 months after the last administration of radium-223, and the occurrence of all post-treatment grade 3/4 haematological toxicities up to 6 months after last administration of radium-223 as adverse events or SAEs.

An adverse event was considered to be serious if it: resulted in death; was life threatening; required hospitalisation (unless the admission resulted in a hospital stay of ≤12 h, or the admission was pre-planned); resulted in persistent or significant disability or incapacity, resulted in a congenital anomaly or birth defect in an offspring; was medically important. A drug-related adverse event was considered as any adverse event judged by the treating physician or radium-223-administering physician (if applicable) as having a reasonable suspected causal relationship to radium-223. In this report, relatedness to study drug (causality assessment) reflected the opinion provided by the reporting investigator.

### Statistics

The REASSURE study aimed to enrol 1200 evaluable patients overall. The first safety interim analysis was planned after the first 600 patients had been enrolled. On 15 February 2016, 602 patients had been enrolled and were identified as patients for the interim analysis. To allow for an approximate 6 months of follow-up, the data cut-off for the interim analysis was 22 September 2016, and included 583 patients who provided signed informed consent and who had received at least one radium-223 injection.

Descriptive analyses were performed on the following subgroups of patients: the prior chemotherapy group included patients who had previously received and completed chemotherapy (docetaxel and/or cabazitaxel) which ended before starting radium-223; the no prior chemotherapy group included patients who had not previously received any chemotherapy (docetaxel and/or cabazitaxel). These patients may have received chemotherapy during the follow-up period after discontinuation of radium-223.

Concomitant treatment was defined as any possible overlap of the medication(s) during radium-223 administration.

## Results

### Patients

At the time of this interim analysis, the median duration of observation of the 583 eligible patients from radium-223 initiation to end of observation was 7 months (range, 0–20 months). Nineteen of these 583 (3%) patients had possible concomitant treatment with chemotherapy and were thus excluded from the exploratory analysis (Online Resource 1). Therefore, analysis according chemotherapy subgroups was performed on 564 patients: 190 (34%) in the prior chemotherapy group and 374 (66%) in the no prior chemotherapy group. Baseline and disease characteristics of these patients are summarised in Table 1. The median age of patients was 73 years (range, 44–94 years), with 78% having an Eastern Cooperative Oncology Group (ECOG) performance status of 0 or 1. A high proportion of patients who had previously received chemotherapy were enrolled in Europe and Israel (74%), and the rest were enrolled in North America (26%). One hundred and twenty-three of 190 patients (65%) who had previously received and completed chemotherapy and 107 of 374 patients (29%) who had not, had also received and completed abiraterone and/or enzalutamide prior to radium-223.

**Table 1:** Baseline characteristics

Characteristic	Prior chemotherapy *N* = 190	No prior chemotherapy *N* = 374	Total *N* = 564
Age, median [range], years	71 [44–90]	74 (52–94)	73 [44–94]
Region			
Europe and Israel	141 (74)	185 (49)	326 (58)
North America	49 (26)	189 (51)	238 (42)
Time between disease stages, median [range], months			
Diagnosis to castration resistance^a^	27 [0–181]	25 (0–236)	25 [0–236]
Castration resistance to study entry^b^	23 [1–108]	10 (0–147)	14 [0–147]
Bone metastases to study entry^c^	34 [<1–167]	19 (<1–236)	23 [<1–236]
Metastases other than bone^d^	49 (26)	62 (17)	111 (20)
ECOG performance status			
0	39 (21)	119 (32)	158 (28)
1	98 (52)	185 (49)	283 (50)
2	35 (18)	30 (8)	65 (12)
3	6 (3)	13 (3)	19 (3)
missing	12 (6)	27 (7)	39 (7)
Extent of metastatic disease^e^			
Normal/abnormal^f^	7 (4)	10 (3)	17 (3)
< 6	18 (10)	77 (22)	95 (18)
6–20	82 (45)	176 (51)	258 (49)
> 20	47 (26)	53 (15)	100 (19)
Superscan	16 (9)	18 (5)	34 (6)
Missing	12 (7)	11 (3)	23 (4)
Laboratory values, median [Q1–Q3]			
PSA, ng/mL^g^	132.0 [38.9–379.2]	40.2 [9.8–153.5]	61.0 [15.4–241.0]
ALP, U/L^h^	162.0 [90.0–367.0]	115.0 [73.0–233.0]	132.0 [78–277.0]
LDH, U/L^i^	279.0 [196.0–405.0]	262.0 [203.0–432.0]	267.4 [200.0–415.0]
Neutrophils ×10^9^/L^j^	4.2 [3.3–6.0]	4.2 [3.0–5.7]	4.2 [3.1–5.7]
Platelets × 10^9^/L^k^	236 [197.0–301.0]	233 [190.0–280.0]	233 [193.0–289.0]
Haemoglobin, g/dL^l^	11.8 [10.4–13.0]	12.3 [11.3–13.3]	12.1 [11.0–13.2]
Prior completed use of abiraterone/enzalutamide	123 (65)	107 (29)	230 (41)

Data are *n* (%) unless otherwise stated

*ALP* alkaline phosphatase, *ECOG* Eastern Cooperative Oncology Group, *LDH* lactate dehydrogenase, *PSA* prostate-specific antigen

^a^Total patients *n* = 234, prior chemotherapy *n* = 84, no prior chemotherapy *n* = 150

^b^Total patients *n* = 280, prior chemotherapy *n* = 100, no prior chemotherapy *n* = 180

^c^Total patients *n* = 391, prior chemotherapy *n* = 138, no prior chemotherapy *n* = 253

^d^Including soft tissue progression in bone (epidural), locoregional expansion (rectum/urethra), visceral metastases (lung, kidney, liver, thyroid and adrenal gland) and distant lymph nodes

^e^Total patients *n* = 527, prior chemotherapy *n* = 182, no prior chemotherapy *n* = 345. For patients with multiple baseline assessments, the worst value is considered. The % base is the number of patients for whom bone scan has been documented at respective visit

^f^Normal or abnormal due to benign bone disease

^g^Total patients *n* = 395, prior chemotherapy *n* = 150, no prior chemotherapy *n* = 245

^h^Total patients *n* = 399, prior chemotherapy *n* = 145, no prior chemotherapy *n* = 254

^i^Total patients *n* = 200, prior chemotherapy *n* = 85, no prior chemotherapy *n* = 115

^j^Total patients *n* = 372, prior chemotherapy *n* = 138, no prior chemotherapy *n* = 234

^k^Total patients *n* = 487, prior chemotherapy *n* = 169, no prior chemotherapy *n* = 318

^l^Total patients *n* = 497, prior chemotherapy *n* = 172, no prior chemotherapy *n* = 325

Patients who had been treated with chemotherapy prior to radium-223 appeared to have poorer baseline characteristics than those who had not (Table 1). This included a higher proportion of patients with an ECOG performance status of 2 or 3 (22% vs 11%), a higher proportion with >20 metastatic lesions (not including superscan), as observed on bone scans (26% vs 15%), and higher median levels of prostate-specific antigen (PSA, 132.0 vs 40.2 ng/mL) and alkaline phosphatase (ALP, 162.0 vs 115.0 U/L), in the group who had previously received chemotherapy compared with those who had not. In addition, patients previously treated with chemotherapy had longer median times with castration-resistant disease (23 vs 10 months) and with bone metastases (34 vs 19 months) before study entry.

Eighty-five of 190 patients (45%) who had received chemotherapy prior to radium-223, and 237 of 374 patients (63%) who had not previously received chemotherapy, had completed six radium-223 injections; the median number of radium-223 injections was five and six, respectively, in these groups (Table 2). Fifty-nine (31%) patients who had previously received chemotherapy and 148 (40%) who had not, were treated with concomitant abiraterone and/or enzalutamide.

**Table 2:** Summary of radium-223 treatment exposure

	Prior chemotherapy *N* = 190	No prior chemotherapy *N* = 374	Total *N* = 564
Number of injections			
Median [Q1–Q3]	5 [3–6]	6 [4–6]	6 [3–6]
Number completed			
1–3	65 (34)	85 (23)	150 (27)
4	22 (12)	31 (8)	53 (9)
5	18 (9)	21 (6)	39 (7)
6	85 (45)	237 (63)	322 (57)
Duration of treatment, median [range], months	3.7 [<0.1–7.2]	4.6 [<0.1–6.7]	4.6 [<0.1–7.2]

### Safety

Overall safety data are summarised in Table 3. In total, 300 of 564 patients (53%) experienced any adverse event. Drug-related TEAEs were reported in 213 patients (38%), the most common of which were gastrointestinal disorders in 118 patients (21%) and haematological toxicities in 73 patients (13%). Drug-related TEAEs were recorded in 78 of 190 (41%) patients who had received prior chemotherapy and 135 of 374 (36%) patients who had not; most of these were gastrointestinal disorders, which occurred in 41 (22%) and 77 (21%) patients, respectively. Haematological toxicities were reported in 39 of 190 (21%) patients who had received prior chemotherapy and 34 of 374 (9%) patients who had not.

**Table 3:** Summary of safety data

	Prior chemotherapy *N* = 190	No prior chemotherapy *N* = 374	Total *N* = 564
Any AE	119 (63)	181 (48)	300 (53)
Drug-related TEAE	78 (41)	135 (36)	213 (38)
Worst grade ≥ 3	25 (13)	25 (7)	50 (9)
Leading to permanent discontinuation of radium-223	17 (9)	17 (5)	34 (6)
Treatment-emergent SAE	66 (35)	73 (20)	139 (25)
Leading to hospitalization or prolongation of existing hospitalization	55 (29)	63 (17)	118 (21)
Leading to permanent discontinuation of radium-223	31 (16)	37 (10)	68 (12)
Leading to death	24 (13)	12 (3)	36 (6)
Drug-related SAE	13 (7)	12 (3)	25 (4)
Leading to permanent discontinuation of radium-223	4 (2)	5 (1)	9 (2)
Leading to death	4 (2)	0	4 (<1)

*AE* adverse event, *SAE* serious adverse event, *TEAE* treatment-emergent adverse event

Drug-related TEAEs by MedDRA preferred term and worst NCI-CTCAE grade occurring in ≥3% of patients in any treatment group or selected for relevance for chemotherapy are summarized in Table 4. Differences in the observed frequency of drug-related TEAEs by MedDRA preferred term of any grade between patients who had received previous chemotherapy and those who had not, included anaemia (15% vs 7%), leukopenia (3% vs 1%), thrombocytopenia (5% vs 3%), and nausea (13% vs 9%). The most common grade 3 or 4 drug-related TEAE was anaemia, which was reported in 23 of 564 patients (4%), occurring in 15 of 190 patients (8%) who had received prior chemotherapy and 8 of 374 (2%) who had not. Grade 3 or 4 fractures in the prior chemotherapy group included a femoral neck fracture, a hip fracture, and a spinal compression fracture, and in the no prior chemotherapy group included a femur fracture, a hip fracture, and a humerus fracture (Online Resource 2).

**Table 4:** Drug-related treatment-emergent adverse events by MedDRA preferred term and NCI-CTCAE worst grade

TEAE	Prior chemotherapy *N* = 190		No prior chemotherapy *N* = 374		Total *N* = 564	
	Any grade	Grade 3 or 4	Any grade	Grade 3 or 4	Any grade	Grade 3 or 4
At least one TEAE	78 (41)^a^	22 (12)	135 (36)^b^	25 (6)	213 (38)^c^	47 (8)
Haematological						
At least one event^d^	39 (21)	16 (8)	34 (9)	16 (4)	73 (13)	32 (6)
Anaemia^e^	28 (15)	15 (8)	27 (7)	8 (2)	55 (10)	23 (4)
Thrombocytopenia^f^	9 (5)	3 (2)	10 (3)	3 (1)	19 (3)	6 (1)
Leukopenia^g^	5 (3)	0	3 (1)	1 (<1)	8 (1)	1 (<1)
Lymphopenia^h^	1 (<1)	0	4 (<1)	1 (<1)	5 (<1)	1 (<1)
Neutropenia^i^	3 (2)	0	3 (<1)	2 (<1)	6 (1)	2 (<1)
Pancytopenia	3 (2)	0	3 (1)	2 (<1)	6 (1)	2 (<1)
Bone marrow failure	2 (1)	0	1 (<1)	1 (<1)	3 (<1)	1 (<1)
Non haematological						
Diarrhoea	19 (10)	0	46 (12)	2 (<1)	65 (12)	2 (<1)
Nausea	24 (13)	0	33 (9)	0	57 (10)	0
Vomiting	8 (4)	0	14 (4)	0	22 (4)	0
Fatigue	14 (7)	2 (1)	29 (8)	1 (<1)	43 (8)	3 (<1)

Data are number of patients (%) reported by MedDRA preferred terms and NCI-CTCAE worst grade in ≥3% of patients in any treatment group or selected for relevance to chemotherapy and radium-223. Drug-related TEAEs: up to 30 days after last radium-223 administration

*MedDRA* Medical Dictionary for Regulatory Activities version 19. *TEAE* treatment-emergent adverse event

^a^Includes six patients where data are missing

^b^Includes two patients where data are missing

^c^Includes eight patients where data are missing

^d^Includes anaemia^e^, thrombocytopenia^f^, leukopenia^g^, lymphopenia^h^, neutropenia^i^, pancytopenia, and bone marrow failure

^e^Anaemia is defined by MedDRA preferred terms: Anaemia, Haemoglobin decreased, and Red blood cell count decreased

^f^Thrombocytopenia is defined by MedDRA preferred terms: Thrombocytopenia and Platelet count decreased

^g^Leukopenia is defined by MedDRA preferred terms: Leukopenia and White blood cell count decreased

^h^Lymphopenia is defined by MedDRA preferred term: Lymphopenia and Lymphocyte count decreased

^i^Neutropenia is defined by MedDRA preferred terms: Neutropenia and Neutrophil count decreased

The incidence of treatment discontinuation due to treatment-emergent drug-related adverse events was low, occurring in 34 of 564 patients (6%) overall, including 17 of 190 (9%) who had previously been treated with chemotherapy, and 17 of 374 (5%) patients who had not (Table 3).

Drug-related SAEs occurred in 13 of 190 (7%) patients who had previously received chemotherapy and 12 of 374 (3%) who had not, the most common of which were haematological toxicities reported in eight and five patients, respectively.

Post-treatment grade 3 or 4 bone marrow suppression events were reported in 50 of 564 patients (9%), occurring in 24 of 190 (13%) who had received prior chemotherapy and in 26 of 374 (7%) who had not (Online Resource 3). The most common were haematopoietic erythropenia in 11% vs 6% and thrombocytopenia in 4% vs 2% of patients who had or had not previously received chemotherapy.

Blood transfusions were received prior to radium-223 therapy in 46 of 546 patients (8%) and concomitantly with radium-223 in 68 patients (12%), including 21 (4%) who received transfusions both before and during radium-223 therapy. Patients who had received prior chemotherapy were more likely to undergo blood transfusions than those who had not, either prior to radium-223 (17% vs 5%) or concomitantly with radium-223 (21% vs 8%) (Table 5).

**Table 5:** Blood transfusions

	Prior chemotherapy *N* = 190	No prior chemotherapy *N* = 374	Total *N* = 564
Prior to radium-223	32 (17)	14 (5)	46 (8)
Concomitant with radium-223	39 (21)	29 (8)	68 (12)
Both prior to and concomitant with radium-223	17 (9)	4 (1)	21 (4)

Data presented are *n* (%)

There were four reported drug-related SAEs leading to death, all occurring in patients who had received prior chemotherapy. These included one patient each with anaemia, pancytopenia, and monocytic leukaemia, and one patient where the event was not reported.

## Discussion

The data from the first interim analysis of the REASSURE study demonstrated that radium-223 had a good short-term safety profile in patients with mCRPC with bone metastases treated in routine clinical practice across the European Union, Israel, and the United States. These findings are in line with the safety profiles previously reported for radium-223 in patients with bone mCRPC in the clinical trial setting [1, 9, 10]. Compared with the ALSYMPCA study [1], patients in the present analysis tended to have less advanced disease, as evidenced by fewer metastatic lesions (≤20 lesions in 67% vs 59% and superscan in 6% vs 9%) and lower median levels of PSA (61 ng/mL vs 146 ng/mL) and ALP (132 U/L vs 211 U/L).

For many years, docetaxel was the only systemic treatment option with a proven survival benefit for mCRPC [5, 6]. However, more recently, abiraterone, enzalutamide, sipuleucel-T (only in the United States), cabazitaxel, and radium-223 are survival-prolonging treatment options for patients with mCRPC [4, 11]. The optimal patient profile for radium-223, and its best use in sequence with the other approved agents, however, is unclear, with no level 1 evidence reported and few consensus opinions available. In the present analysis, patients who had not previously received chemotherapy appeared to have less advanced disease than patients who had received prior chemotherapy, as suggested by their more favourable baseline disease characteristics, including a shorter time for which tumours had been castration-resistant, fewer patients with an ECOG performance status ≥2, the presence of fewer metastatic lesions, and lower baseline PSA and ALP levels. In addition, patients who had not previously received chemotherapy more often completed six radium-223 injections than those who had received prior chemotherapy. Similarly, completion of radium-223 was reported to be more likely in patients with less advanced disease (as defined by better baseline characteristics) treated in a community-based setting [12, 13]. Furthermore, the better safety profile and lower rates of treatment discontinuation reported in patients who had not previously received chemotherapy compared with those who had, might also suggest that patients with mCRPC and bone metastases may benefit from radium-223 earlier in their treatment course, before administration of chemotherapy. Alternatively, this may simply reflect incomplete recovery from the prior effects of chemotherapy in the previously treated patient group.

Bone marrow function is often compromised in patients with bone metastases [14]; therefore, assessing bone marrow suppression and the need for bone-targeting treatments is of interest in this patient group. In contrast to radiopharmaceuticals that emit long-range beta particles, the short-range alpha particles emitted by radium-223 theoretically result in a more localised effect, with less bone marrow suppression and fewer related adverse events [15, 16]. In the current post hoc analysis, blood transfusions were received by 46 patients (8%) prior to initiation of radium-223 and by 68 patients (12%) during the course of radium-223 therapy; 21 of these patients received transfusions both before and during radium-223 therapy. Notably, patients who had received prior chemotherapy were more likely to have received blood transfusions prior to receiving radium-223, or concomitantly with radium-223. In the ALSYMPCA study, 42% of patients required blood transfusions from randomisation to study end. Post hoc analyses from the ALSYMPCA study identified significant baseline predictors for haematological toxicity related to radium-223 treatment, which included the extent of bone disease, PSA levels, decreased haemoglobin levels and platelet counts, and prior chemotherapy use [17]. The authors recommended that such factors should be considered in the management of patients with mCRPC treated with radium-223.

In this analysis, drug-related haematological SAEs occurred more frequently in patients with a history of prior chemotherapy use; these patients generally had a higher burden of bone disease and more often discontinued treatment, suggesting that the higher haematological adverse event rate was perhaps a consequence of prior treatment and/or disease stage rather than radium-223-related toxicity. Subsequent analyses of the REASSURE study may help clarify the long-term effects of radium-223 on bone marrow. Safety studies such as this play a critical role in helping to determine the optimal treatment approach for patients.

This study provides the opportunity to prospectively collect safety data on radium-223 in routine clinical practice, although caution must be used in interpreting the results due to the observational nature of the study. In particular, the single-arm study design does not allow comparison with a control group. The current analysis is of particular interest, since radium-223 is commonly administered later in the disease course, often after chemotherapy and in patients that are considered not suitable for chemotherapy or where chemotherapy is contraindicated [1, 18, 19]. Some physicians consider that it is important for management of the disease that the opportunity is provided to administer as many of the available life-prolonging therapies as possible. Therefore, it may be the case that radium-223 might be better placed early in the disease course during initial lines of treatment, but must be placed prior to the onset of visceral disease (lung, liver, or other organ metastases) [20].

Although the short follow-up time for this interim analysis precluded data on overall survival, subgroup analyses from the ALSYMPCA trial showed an overall survival benefit for radium-223 irrespective of prior docetaxel use [7]. Exploratory analyses suggested that administration of docetaxel following radium-223 was viable, well tolerated, and did not adversely affect overall survival [8]. Long-term safety and outcomes on all patients in REASSURE will be reported in the next interim analysis, expected in 2019.

In conclusion, the short-term safety profile of radium-223, when used in routine clinical practice settings in patients with mCRPC with bone metastases, was comparable to other clinical studies, regardless of prior chemotherapy use, with no unexpected findings reported. Drug-related SAEs were most often haematological and occurred more frequently in patients with a history of prior chemotherapy use and a higher burden of disease, which could potentially be due to advanced disease or toxicity from prior chemotherapy. Patients who had not previously received chemotherapy appeared to have a lower burden of disease at baseline, experienced a lower incidence of haematological adverse events, and discontinued radium-223 treatment less often. These results may suggest that selected patients with mCRPC with bone metastases may benefit from the use of radium-223 earlier in the course of treatment, before treatment with chemotherapy.

## Electronic supplementary material

Online Resource 1 (DOCX 31 kb)

Online Resource 2 (DOCX 28 kb)

Online Resource 3 (DOCX 28 kb)
