# Supplementary material for: Interim analysis of the REASSURE (Radium-223 alpha Emitter Agent in non-intervention Safety Study in mCRPC popUlation for long-teRm Evaluation) study: patient characteristics and safety according to prior use of chemotherapy in routine clinical practice
Source: Eur J Nucl Med Mol Imaging. 2019 Jan 12;46(5):1102–10. doi: 10.1007/s00259-019-4261-y (PMC6451709; doi:10.1007/s00259-019-4261-y)
Supplement: Supplementary file 1 — (DOCX 31 kb) [file 259_2019_4261_MOESM1_ESM.docx]

**Interim analysis of the REASSURE (Radium-223 alpha Emitter Agent in non-intervention Safety Study in mCRPC popUlation for long-teRm Evaluation) study: patient characteristics and safety according to prior use of chemotherapy in routine clinical practice**

Sabina Dizdarevic, Peter Meidahl Petersen, Markus Essler, Annibale Versari,
Jean-Cyril Bourre, Christian la Fougère, Riccardo Valdagni, Giovanni Paganelli,
Samer Ezziddin, Ján Kalinovský, Inga Bayh, Yong Du

**Journal:** European Journal of Nuclear Medicine and Molecular Imaging

**Corresponding author**

Sabina Dizdarevic

Department of Imaging and Nuclear Medicine

Royal Sussex County Hospital

Brighton and Sussex University Hospitals NHS Trust

Eastern Road

Brighton BN2 5BE

Email sabina.dizdarevic@bsuh.nhs.uk

**Online Resource 1** Profile of study analysis populations

^a^Concomitant treatment was defined as any possible overlap of chemotherapy during radium-223 administration. Patients with a start date prior to first radium-223 dose and a missing stop date were considered in this group.

^b^Prior completed treatment group included patients who received chemotherapy started and stopped (with an available stop date) before the first radium-223 injection.

^c^No prior chemotherapy group included patients who did not receive any chemotherapy throughout the observation period (chemo-naive) and those who only received chemotherapy during follow-up after discontinuation of radium-223.

Chemotherapy was defined as docetaxel and or cabazitaxel only.
