# Supplementary material for: Interim analysis of the REASSURE (Radium-223 alpha Emitter Agent in non-intervention Safety Study in mCRPC popUlation for long-teRm Evaluation) study: patient characteristics and safety according to prior use of chemotherapy in routine clinical practice
Source: Eur J Nucl Med Mol Imaging. 2019 Jan 12;46(5):1102–10. doi: 10.1007/s00259-019-4261-y (PMC6451709; doi:10.1007/s00259-019-4261-y)
Supplement: Supplementary file 2 — (DOCX 28 kb) [file 259_2019_4261_MOESM2_ESM.docx]

**Interim analysis of the REASSURE (Radium-223 alpha Emitter Agent in non-intervention Safety Study in mCRPC popUlation for long-teRm Evaluation) study: patient characteristics and safety according to prior use of chemotherapy in routine clinical practice**

Sabina Dizdarevic, Peter Meidahl Petersen, Markus Essler, Annibale Versari,
Jean-Cyril Bourre, Christian la Fougère, Riccardo Valdagni, Giovanni Paganelli,
Samer Ezziddin, Ján Kalinovský, Inga Bayh, Yong Du

**Journal:** European Journal of Nuclear Medicine and Molecular Imaging

**Corresponding author**

Sabina Dizdarevic

Department of Imaging and Nuclear Medicine

Royal Sussex County Hospital

Brighton and Sussex University Hospitals NHS Trust

Eastern Road

Brighton BN2 5BE

Email [sabina.dizdarevic@bsuh.nhs.uk](mailto:sabina.dizdarevic@bsuh.nhs.uk)

**Online Resource 2** Summary of fractures

| **Preferred Term** | **Prior chemotherapy**  **N=190** | | **No prior chemotherapy**  **N=374** | | **Total**  **N=564** | |
| --- | --- | --- | --- | --- | --- | --- |
|  | **Any grade** | **Grade 3 or 4** | **Any grade** | **Grade 3 or 4** | **Any grade** | **Grade 3 or 4** |
| Ankle fracture | 0 | 0 | 1 (<1)* | 0 | 1 (<1)* | 0 |
| Clavicle fracture | 1 (<1) | 0 | 0 | 0 | 1 (<1) | 0 |
| Femoral neck fracture | 1 (<1) | 1 (<1) | 0 | 0 | 1 (<1) | 1 (<1) |
| Femur fracture | 0 | 0 | 2 (<1) | 1 (<1) | 2 (<1) | 1 (<1) |
| Fracture | 1 (<1)* | 0 | 0 | 0 | 1 (<1)* | 0 |
| Hip fracture | 1 (<1) | 1 (<1) | 1 (<1) | 1 (<1) | 2 (<1) | 2 (<1) |
| Humerus fracture | 1 (<1)* | 0 | 2 (<1)* | 0 | 3 (<1)^†^ | 0 |
| Spinal compression fracture | 2 (1) | 1 (<1) | 0 | 0 | 2 (<1) | 1 (<1) |
| Tibia fracture | 0 | 0 | 1 (<1) | 1 (<1) | 1 (<1) | 1 (<1) |
| Tooth fracture | 0 | 0 | 1 (<1) | 0 | 1 (<1) | 0 |
| Upper limb fracture | 1 (<1) | 0 | 0 | 0 | 1 (<1) | 0 |

Data are n (%).

^a^Missing data for 1 patient.

^b^Missing data in 2 patients.
