# Supplementary material for: Interim analysis of the REASSURE (Radium-223 alpha Emitter Agent in non-intervention Safety Study in mCRPC popUlation for long-teRm Evaluation) study: patient characteristics and safety according to prior use of chemotherapy in routine clinical practice
Source: Eur J Nucl Med Mol Imaging. 2019 Jan 12;46(5):1102–10. doi: 10.1007/s00259-019-4261-y (PMC6451709; doi:10.1007/s00259-019-4261-y)
Supplement: Supplementary file 3 — (DOCX 28 kb) [file 259_2019_4261_MOESM3_ESM.docx]

**Interim analysis of the REASSURE (Radium-223 alpha Emitter Agent in non-intervention Safety Study in mCRPC popUlation for long-teRm Evaluation) study: patient characteristics and safety according to prior use of chemotherapy in routine clinical practice**

Sabina Dizdarevic, Peter Meidahl Petersen, Markus Essler, Annibale Versari,
Jean-Cyril Bourre, Christian la Fougère, Riccardo Valdagni, Giovanni Paganelli,
Samer Ezziddin, Ján Kalinovský, Inga Bayh, Yong Du

**Journal:** European Journal of Nuclear Medicine and Molecular Imaging

**Corresponding author**

Sabina Dizdarevic

Department of Imaging and Nuclear Medicine

Royal Sussex County Hospital

Brighton and Sussex University Hospitals NHS Trust

Eastern Road

Brighton BN2 5BE

Email sabina.dizdarevic@bsuh.nhs.uk

**Online Resource 3.** Post treatment grade 3 or 4 haematological adverse events based on bone marrow suppression by worst grade

| **BMS category** | **Prior chemotherapy**  **N=190** | **No prior chemotherapy**  **N=374** | **Total**  **N=564** |
| --- | --- | --- | --- |
| Any BMS relevant event | 24 (13) | 26 (7) | 50 (9) |
| Leukopenia not further specified^a^ | 0 | 0 | 0 |
| Neutropenia^b^ | 0 | 1 (<1) | 1 (<1) |
| Pancytopenia^c^ | 0 | 3 (1) | 3 (<1) |
| Thrombocytopenia^d^ | 7 (4) | 6 (2) | 13 (2) |
| Haematopoietic erythropenia^e^ | 21 (11) | 21 (6) | 42 (7) |

Data are n (%).Post treatment grade 3 o 4 haematological AEs: up to 6 months after last radium-223 administration. Coded using Medical Dictionary for Regulatory Activities (MedDRA) version 19 and graded according to the Common Terminology Criteria for Adverse Events version 4.03.

*BMS* bone marrow suppression.

^a^Leukopenia is defined by the MedDRA preferred terms: Leukopenia and White blood cell count decreased.

^b^Neutropenia is defined by the MedDRA preferred terms: Band neutrophil count decreased, Band neutrophil percentage decreased, Granulocyte count decreased, Granulocytopenia, Idiopathic neutropenia, Neutropenia, Neutrophil count decreased, and [Neutrophil percentage decreased](http://by-gcoding.de.bayer.cnb/mpcprd/mp_browser_meddra.showdetails?i_thesaurus=TH_MEDDRA_PROD&i_user=MPLOGIN&i_level=PT&i_code=10052223).

^c^Pancytopenia is defined by the MedDRA preferred terms: Autoimmune pancytopenia, Full blood count decreased, and Pancytopenia.

^d^Thrombocytopenia is defined is defined by the MedDRA preferred terms: Immune thrombocytopenic purpura, Platelet count decreased, Platelecrit decreased, and Thrombocytopenia.

^e^Haematopoietic erythropenia is defined by the MedDRA preferred terms: [Anaemia](http://by-gcoding.de.bayer.cnb/mpcprd/mp_browser_meddra.showdetails?i_thesaurus=TH_MEDDRA_PROD&i_user=MPLOGIN&i_level=PT&i_code=10002034), [Anaemia macrocytic](http://by-gcoding.de.bayer.cnb/mpcprd/mp_browser_meddra.showdetails?i_thesaurus=TH_MEDDRA_PROD&i_user=MPLOGIN&i_level=PT&i_code=10002064), [Anaemia neonatal](http://by-gcoding.de.bayer.cnb/mpcprd/mp_browser_meddra.showdetails?i_thesaurus=TH_MEDDRA_PROD&i_user=MPLOGIN&i_level=PT&i_code=10002068), [Aplasia pure red cell](http://by-gcoding.de.bayer.cnb/mpcprd/mp_browser_meddra.showdetails?i_thesaurus=TH_MEDDRA_PROD&i_user=MPLOGIN&i_level=PT&i_code=10002965), [Aplastic anaemia](http://by-gcoding.de.bayer.cnb/mpcprd/mp_browser_meddra.showdetails?i_thesaurus=TH_MEDDRA_PROD&i_user=MPLOGIN&i_level=PT&i_code=10002967), [Erythroblast count abnormal](http://by-gcoding.de.bayer.cnb/mpcprd/mp_browser_meddra.showdetails?i_thesaurus=TH_MEDDRA_PROD&i_user=MPLOGIN&i_level=PT&i_code=10058508), [Erythroblast count decreased](http://by-gcoding.de.bayer.cnb/mpcprd/mp_browser_meddra.showdetails?i_thesaurus=TH_MEDDRA_PROD&i_user=MPLOGIN&i_level=PT&i_code=10058505), [Erythroid maturation arrest](http://by-gcoding.de.bayer.cnb/mpcprd/mp_browser_meddra.showdetails?i_thesaurus=TH_MEDDRA_PROD&i_user=MPLOGIN&i_level=PT&i_code=10015279), [Erythropenia](http://by-gcoding.de.bayer.cnb/mpcprd/mp_browser_meddra.showdetails?i_thesaurus=TH_MEDDRA_PROD&i_user=MPLOGIN&i_level=PT&i_code=10015287), [Erythropoiesis abnormal](http://by-gcoding.de.bayer.cnb/mpcprd/mp_browser_meddra.showdetails?i_thesaurus=TH_MEDDRA_PROD&i_user=MPLOGIN&i_level=PT&i_code=10049467), Foetal anaemia, [Haematocrit abnormal](http://by-gcoding.de.bayer.cnb/mpcprd/mp_browser_meddra.showdetails?i_thesaurus=TH_MEDDRA_PROD&i_user=MPLOGIN&i_level=PT&i_code=10049221), [Haematocrit decreased](http://by-gcoding.de.bayer.cnb/mpcprd/mp_browser_meddra.showdetails?i_thesaurus=TH_MEDDRA_PROD&i_user=MPLOGIN&i_level=PT&i_code=10018838), [Haemoglobin abnormal](http://by-gcoding.de.bayer.cnb/mpcprd/mp_browser_meddra.showdetails?i_thesaurus=TH_MEDDRA_PROD&i_user=MPLOGIN&i_level=PT&i_code=10018879), [Haemoglobin decreased](http://by-gcoding.de.bayer.cnb/mpcprd/mp_browser_meddra.showdetails?i_thesaurus=TH_MEDDRA_PROD&i_user=MPLOGIN&i_level=PT&i_code=10018884), [Hypoplastic anaemia](http://by-gcoding.de.bayer.cnb/mpcprd/mp_browser_meddra.showdetails?i_thesaurus=TH_MEDDRA_PROD&i_user=MPLOGIN&i_level=PT&i_code=10021074), [Leukoerythroblastic anaemia](http://by-gcoding.de.bayer.cnb/mpcprd/mp_browser_meddra.showdetails?i_thesaurus=TH_MEDDRA_PROD&i_user=MPLOGIN&i_level=PT&i_code=10053199), [Microcytic anaemia](http://by-gcoding.de.bayer.cnb/mpcprd/mp_browser_meddra.showdetails?i_thesaurus=TH_MEDDRA_PROD&i_user=MPLOGIN&i_level=PT&i_code=10027538), [Normochromic normocytic anaemia](http://by-gcoding.de.bayer.cnb/mpcprd/mp_browser_meddra.showdetails?i_thesaurus=TH_MEDDRA_PROD&i_user=MPLOGIN&i_level=PT&i_code=10029783), [Proerythroblast count abnormal](http://by-gcoding.de.bayer.cnb/mpcprd/mp_browser_meddra.showdetails?i_thesaurus=TH_MEDDRA_PROD&i_user=MPLOGIN&i_level=PT&i_code=10060227), [Proerythroblast count decreased](http://by-gcoding.de.bayer.cnb/mpcprd/mp_browser_meddra.showdetails?i_thesaurus=TH_MEDDRA_PROD&i_user=MPLOGIN&i_level=PT&i_code=10060229), [Red blood cell count abnormal](http://by-gcoding.de.bayer.cnb/mpcprd/mp_browser_meddra.showdetails?i_thesaurus=TH_MEDDRA_PROD&i_user=MPLOGIN&i_level=PT&i_code=10038151), [Red blood cell count decreased](http://by-gcoding.de.bayer.cnb/mpcprd/mp_browser_meddra.showdetails?i_thesaurus=TH_MEDDRA_PROD&i_user=MPLOGIN&i_level=PT&i_code=10038153), [Reticulocyte count abnormal](http://by-gcoding.de.bayer.cnb/mpcprd/mp_browser_meddra.showdetails?i_thesaurus=TH_MEDDRA_PROD&i_user=MPLOGIN&i_level=PT&i_code=10038788), [Reticulocyte count decreased](http://by-gcoding.de.bayer.cnb/mpcprd/mp_browser_meddra.showdetails?i_thesaurus=TH_MEDDRA_PROD&i_user=MPLOGIN&i_level=PT&i_code=10038790), [Reticulocyte percentage decreased](http://by-gcoding.de.bayer.cnb/mpcprd/mp_browser_meddra.showdetails?i_thesaurus=TH_MEDDRA_PROD&i_user=MPLOGIN&i_level=PT&i_code=10059921), [Reticulocytopenia](http://by-gcoding.de.bayer.cnb/mpcprd/mp_browser_meddra.showdetails?i_thesaurus=TH_MEDDRA_PROD&i_user=MPLOGIN&i_level=PT&i_code=10038795).
